# Submergence Stress Alters the Expression of Clock Genes and Configures New Zeniths and Expression of Outputs in *Brachypodium distachyon*

**DOI:** 10.3390/ijms24108555

**Published:** 2023-05-10

**Authors:** Lucisabel Medina-Chávez, Christian Camacho, Jorge Arturo Martínez-Rodríguez, Blanca Estela Barrera-Figueroa, Dawn H. Nagel, Piyada Juntawong, Julián Mario Peña-Castro

**Affiliations:** 1 Centro de Investigaciones Científicas, Instituto de Biotecnología, Universidad del Papaloapan, Tuxtepec 68301, Oaxaca, Mexico; 2 Programa de Doctorado en Biotecnología, División de Estudios de Posgrado, Universidad del Papaloapan, Tuxtepec 68301, Oaxaca, Mexico; 3 Department of Botany and Plant Sciences, University of California, Riverside, CA 92521, USA; 4 Laboratorio de Biotecnología Vegetal, Instituto de Biotecnología, Universidad del Papaloapan, Tuxtepec 68301, Oaxaca, Mexico; 5 Department of Genetics, Faculty of Science, Kasetsart University, Bangkok 10900, Thailand; 6 Omics Center for Agriculture, Bioresources, Food and Health, Kasetsart University (OmiKU), Bangkok 10900, Thailand

**Keywords:** hypoxia, submergence, circadian rhythm, diurnal expression, RNA-seq, chronoculture, abiotic stress

## Abstract

Plant networks of oscillating genes coordinate internal processes with external cues, contributing to increased fitness. We hypothesized that the response to submergence stress may dynamically change during different times of the day. In this work, we determined the transcriptome (RNA sequencing) of the model monocotyledonous plant, *Brachypodium distachyon*, during a day of submergence stress, low light, and normal growth. Two ecotypes of differential tolerance, Bd21 (sensitive) and Bd21-3 (tolerant), were included. We submerged 15-day-old plants under a long-day diurnal cycle (16 h light/8 h dark) and collected samples after 8 h of submergence at ZT0 (dawn), ZT8 (midday), ZT16 (dusk), ZT20 (midnight), and ZT24 (dawn). Rhythmic processes were enriched both with up- and down-regulated genes, and clustering highlighted that the morning and daytime oscillator components (*PRRs*) show peak expression in the night, and a decrease in the amplitude of the clock genes (*GI*, *LHY*, *RVE*) was observed. Outputs included photosynthesis-related genes losing their known rhythmic expression. Up-regulated genes included oscillating suppressors of growth, hormone-related genes with new late zeniths (e.g., *JAZ1*, *ZEP*), and mitochondrial and carbohydrate signaling genes with shifted zeniths. The results highlighted genes up-regulated in the tolerant ecotype such as *METALLOTHIONEIN3* and *ATPase INHIBITOR FACTOR*. Finally, we show by luciferase assays that *Arabidopsis thaliana* clock genes are also altered by submergence changing their amplitude and phase. This study can guide the research of chronocultural strategies and diurnal-associated tolerance mechanisms.

## 1. Introduction

Drought and flooding are the two principal drawbacks that crops face in the field, together comprising >50% of the billions of dollars of yearly agricultural losses [1,2]. Climate science has consistently shown that global warming causes the appearance of hydroclimate wet/dry extremes that will challenge several agriculturally productive areas in decades to come [3]. Damage caused by the flooding of crop fields has deep economic and social effects that increase poverty, vulnerability, and migration [4]. Therefore, the elucidation of the natural plant responses to water stress is of great importance to design technical approaches to maintain agricultural productivity in the future.

Flooding can be divided into two forms of stress depending on the depth of the water column in relation to the height of the plant. The stress is either called waterlogging if only the roots are flooded or submergence if the entire plant is under the water [5]. Both types of flooding lower oxygen diffusion, causing hypoxia to the plant organs and prompting a compound response that continually senses the hypoxic state, remodels the plant metabolism to avoid an over-reduction of the essential NAD^+^/NADH biochemical pool [6], modulates the responses to manage energy reserves, and prepares the plant cells for a probable return to normoxic conditions [7,8].

Some components of these hypoxic stress responses are known outputs of the circadian clock in other contexts, either under normal growth or stress conditions. A classic example of a diurnal and circadian regulated output is starch biosynthesis [9], which is also an essential biochemical base for submergence survival in Arabidopsis (*Arabidopsis thaliana*; [10]). Flowering time is also a diurnal and circadian output, and is molecularly repressed during submergence stress in rice (*Oryza sativa*), Arabidopsis [11], and Brachypodium (*Brachypodium distachyon*; [12]). Hormone synthesis is another output of the circadian regulation; for example, jasmonate and salicylate are diurnally regulated [13] but repressed in Arabidopsis under submergence stress [14]. Conversely, ethylene starts to be diurnally produced in waterlogging-tolerant *Rumex palustris* [15]. Reactive oxygen species (ROS) are regulated with a mixed diurnal and circadian rhythmicity in Arabidopsis [16], and under submergence, the modulated production of ROS is a marker of tolerance in Arabidopsis [17] and in Brachypodium ecotypes of contrasting tolerance [12]. Additionally, in Brachypodium, the transcription of *ALTERNATIVE OXIDASE 1* was diurnally regulated and more abundant in the submergence-sensitive ecotype [12].

Not only the outputs of the circadian clock are affected by oxygen-related stresses; there is evidence that waterlogging [18] and hypoxic stress [19] affect the circadian clock genes’ expression amplitude and/or zeniths (peak expression). *TIME OF CAB EXPRESSION* (*TOC1*) was downregulated in soybean under long-term waterlogging (15d), and *PSEUDO RESPONSE REGULATOR 3* and *9* (*PRR3*, *PRR9*) advanced its phase from midday to dusk [18]. Lee and Bailey-Serres [19] used Arabidopsis seedlings entrained for a long-day photoperiod and imposed severe hypoxic stress (<2% O_2_) during the night, which caused the rapid misregulation of several clock genes (2–9 h after stress), indicating a new configuration of the clock with extended presence of evening and night regulators.

Based on the above observations, we hypothesized that the plant transcriptional response to submergence stress may be dynamically changing according to the pressures faced during different times of the day. In this work, we analyzed the transcriptome of the model monocotyledonous plant, Brachypodium, during a whole day of submergence stress. We studied Brachypodium ecotypes of differential tolerance to propose potential markers and unexplored mechanisms for submergence survival. Finally, we also included Arabidopsis to follow the activity of promoters of clock genes.

## 2. Results

### 2.1. Brachypodium Ecotypes of Differential Submergence Stress Tolerance

**Figure 1:**
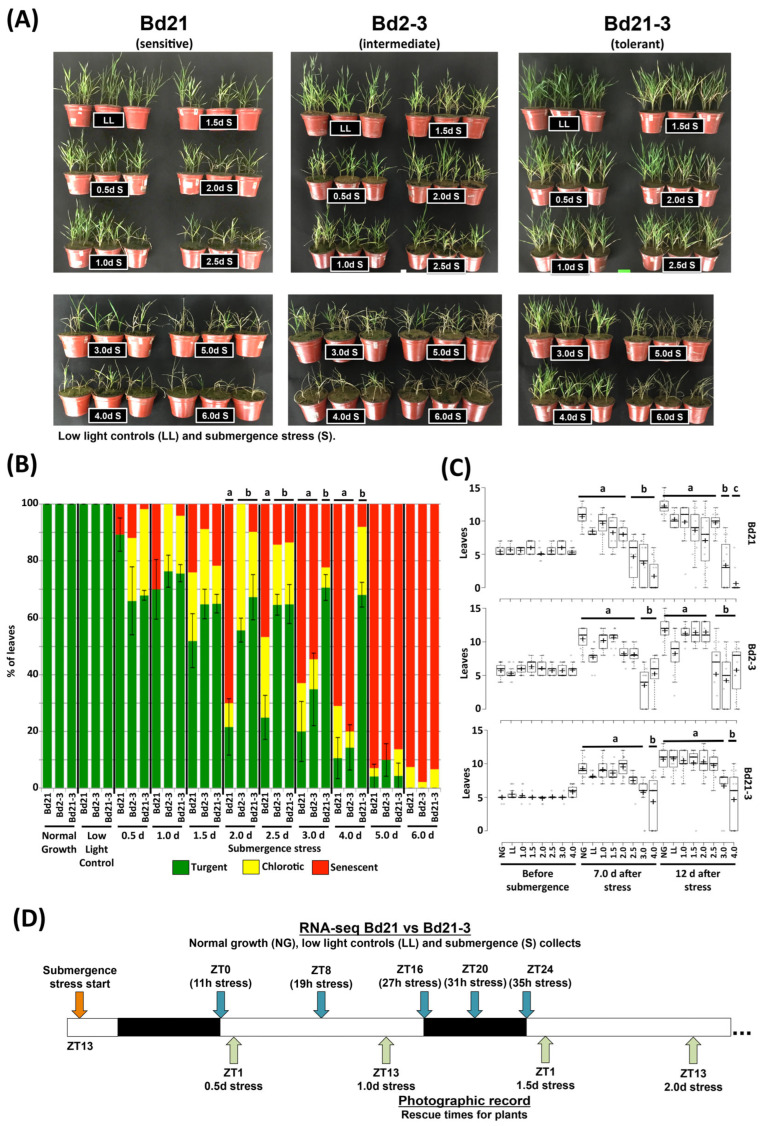
Growth of Brachypodium ecotypes of contrasting submergence tolerance and experimental setup. For panel (**A**–**C**), the photographs and data were collected from plants subjected to submergence stress simultaneously at 14 days old after germination for the indicated times and low light (LL) or normal growth (NG) controls. (**A**) Images of plants 8 days after submergence stress was imposed for the indicated times; all plants are of the same age (22 days old), and their recovery time (Rt) is Rt = 8–X, where X is days under stress. (**B**) Quantification of damaged leaves classified as turgent, chlorotic, or senescent. (**C**) Total number of leaves after recovery time from the indicated submergence stress treatment quantified after 7 d (21 days old) or 12 d (26 days old) of imposing stress. (**D**) Experimental setup of treatment and RNA sequencing sample collection and for images shown in panel **A** and Figure S1. Data are from 3 independent experiments, error bars are SD, *n* = 8–15 plants. Different letters indicate significant differences (*p* < 0.05) indicated by one-way ANOVA with post hoc Tukey HSD.

In a previous work, we screened different Brachypodium ecotypes for submergence stress tolerance and determined that ecotype Bd21 is more sensitive than ecotype Bd2-3 [12]. While other ecotypes exhibit more tolerance to submergence, i.e., Bd1-1 and Tek-10, they express an extended juvenile stage when compared to Bd21 and Bd2-3 [12]. Therefore, we compared ecotypes of similar developmental programs such as Bd21 and Bd2-3. Bd21-3 was included because it is the standard ecotype that has been used to generate the published T-DNA insertion mutant collection [20], and we detected a fit phenotype under submergence in preliminary experiments. When submergence stress was simultaneously applied to these three ecotypes under long-day with low light (LL) conditions, Bd21 showed stress symptoms such as stunted growth, arrested leaf development and chlorosis even after 1 d of submergence, while Bd2-3 plants showed similar symptoms after 2.5 d and Bd21-3 after 4 d of stress (Figure 1A). 

We adapted for Brachypodium the leaf damage index proposed for Arabidopsis that classifies individual plants according to their leaf damage [21]. We observed that Bd21-3 could significantly avoid higher percentages of senescent and chlorotic leaves for up to 4 d of stress when compared to Bd2-3 and Bd21, which displayed these stress symptoms after 3 d and 2 d of stress, respectively (Figure 1B). All ecotypes succumbed after 6 d of stress (Figure 1B).

We quantified the consequences of the stress on the number of green leaves developed after submergence stress (Figure 1C). After 7 d of recovery, Bd21 plants stressed for 2.5 d or more could not develop the same number of leaves as its normal growth (NG; undisturbed) or low light controls (LL; receiving 1/2 intensity of light as NG). After 12 d of recovery, Bd21 plants that were stressed for 3 d had fewer leaves than controls and had irreversible development arrest after 3 d (Figure 1C). The most tolerant ecotype, Bd21-3, showed reversible damage up to 4 d of stress and a more robust response on all stress times applied observed as a compact dispersion of developed leaves per plant (Figure 1C). 

We conclude from these experiments that Bd2-3 is more tolerant to submergence than Bd21 and that Brachypodium expresses a quiescent tolerance strategy. In addition, we now report that Bd21-3 is an ecotype with a more robust response to submergence. 

### 2.2. Brachypodium Transcriptome under Submergence Stress Responds to Time of Day

We selected Bd21 and Bd21-3 as sensitive and tolerant ecotypes to characterize and compare their transcriptomes under submergence stress. For this, we performed the experimental setup shown in Figure 1D, where long-day entrained plants were submerged at ZT13, and then collected during the next day of stress at dawn (ZT0, 11 h of stress), midday (ZT8, 19 h of stress), dusk (ZT16 27 h of stress), midnight (ZT20, 31 h of stress), and the next dawn (ZT24, 35 h of stress). Simultaneously, NG (undisturbed) and LL (low light) controls were collected at the same ZTs for a total of 90 libraries. In parallel, we kept and photographed plants uncollected and subjected to identical experimental conditions to confirm the consistency of the differential response in the sequenced samples (Figure S1). 

**Figure 2:**
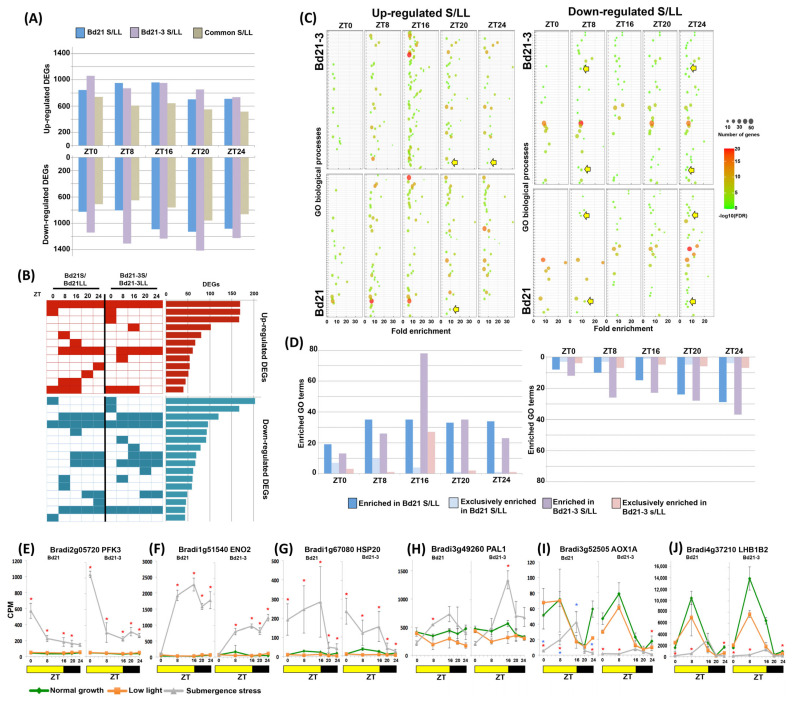
The diurnal transcriptome expressed under submergence stress by Brachypodium ecotypes of contrasting tolerance. (**A**) Number of DEGs at each individual ZT and (**B**) number of DEGs across all ZTs, both exclusive and common to the two contrasting ecotypes when submergence stress is compared to low light controls. (**C**) Biological processes’ GO terms enriched by up- and down-regulated DEGs at each ZT; yellow arrows indicate diurnal/circadian terms (expanded version in Figure S2). (**D**) Number of different biological processes’ GO terms present at each ZT of both up- and down-regulated DEGs. (**E**–**J**) Diurnal expression of selected DEGs from differentially enriched GO terms under submergence stress, normal growth, and low light controls. Data are means of CPM measured in 3 independent RNA sequencing experiments and error bars indicate SD. Red asterisks indicate significant differences (FDR < 5 × 10^−6^) between submergence stress and low light values; blue asterisks indicate significant differences (FDR < 5 × 10^−6^) between submergence stress values of Bd21 and Bd21-3.

Transcriptomic changes were determined by RNA sequencing. The data were analyzed using as a main comparison the submerged samples against the LL controls. Although we observed that the commonly differentially expressed genes (DEGs) by both ecotypes were the majority, each ecotype conserved a distinct/specific subset of DEGs. This was most evident in the down-regulated DEGs where tolerant Bd21-3 had more transcripts than sensitive Bd21 (Figure 2A). 

Since there were no large differences in the total number of DEGs detected at each ZT, we separated the individual DEGs based on the ZT where they showed a transcriptional response to the stress (Figure 2B). A minority of DEGs were up- or down-regulated at every ZT studied, and the majority of DEGs had a specific period of time where they were differential, being ZT0 the most represented, suggesting the detection of a subset of transcripts that can be considered part of an early shock response expressed during the first night of stress. The next ZTs with more DEGs were ZT16 in Bd21-3, followed by ZT8 in Bd21, indicating that there was an adjustment of expression dependent on the time of day. In addition, both ecotypes had groups of DEGs exclusively expressed in complex manners on individual ZTs or in periods of ZTs (Figure 2B; Table S1).

### 2.3. Submergence Stress Changed the Biological Processes Enriched at Each ZT

Next, DEGs were collectively characterized as gene ontology (GO) terms of biological processes. The detected GO terms included some expected and well-known responses to submergence stress such as amino acid metabolic processes, chaperone refolding, and nucleotide and glycolytic metabolic processes, as well as to different stresses (heat, salt, oxidative, drought, and wounding; Table S2). However, we observed that the number of GO terms enriched on specific ZTs, as well as the number of DEGs enriched on each GO term, changed dynamically depending on the time of day (Figure 2C). For example, DEGs up-regulated at ZT16 enriched the largest collection of terms on tolerant Bd21-3 (Figure 2D), including ADP metabolic processes, ethylene stimulus, and the chorismate/benzene-containing compound metabolic process. For Bd21, examples of enriched groups belonged to alternative respiration, glutamate and glutathione metabolism, or response to wounding, confirming our previous observations that the sensitive ecotype expressed more responses to oxidative stress [12]. 

Only four up-regulated GO terms were enriched (fold enrichment ≥ 5) in all five ZTs tested on both ecotypes. These were redundant terms “nucleotide phosphorylation”/”pyruvate metabolic process”, and “protein complex oligomerization”/”response to hydrogen peroxide”. Still, these terms had enriching DEGs with varying diurnal expression when compared to LL controls. The first group was enriched for DEGs coding for glycolytic enzymes and the second by *HEAT SHOCK PROTEINS* (*HSP*). Glycolytic enzymes had patterns of different expression intensities across ZTs. This observation helped us to confirm that our sampling captured transcripts coding for enzymes responding to an initial shock response (e.g., *PHOSPHOFRUCTOKINASE 3*, *PFK3*; Figure 2E) that came out of the night with high transcription tending to stabilize during the light period, and transcripts belonging to a second wave of responses (e.g., *ENOLASE 2*, *ENO2*; Figure 2F) that started at dawn with low transcription and increased their presence as the day advanced. These examples did not show a rhythmic expression throughout the day, and rather showed up-regulated values when compared to control that decreased or increased as the stress continued. This may reflect the existence of dynamic metabolic signals that build up or recede at different stress stages.

*HSP* up-regulated their transcriptional activity during the light period and decreased in the night (Figure 2G). Differential ZT/ecotype-dependent GO terms revealed transcripts that exhibit a significantly higher peak of transcript abundance under submergence such as *PHENYLALANINE LYASE* (*PAL*; Figure 2H) or that showed a shift in transcriptomic zenith to dusk such as *ALTERNATIVE OXIDASE 1A* (*AOX1A*; Figure 2I). *AOX1A* was also part of exclusively enriched terms in sensitive Bd21; the shifted zenith at ZT16 in Bd21 was notably reduced in the tolerant Bd21-3. These genes are known to be under diurnal and/or circadian control in Arabidopsis [16,22,23,24], and interestingly, in Brachypodium under stress, diurnal expressions either gained apparent rhythmicity (*PAL*), changed peak accumulation (*AOX1A*), or were responsive to the stress advancement (*HSP20*). Taken together, GO term enrichment indicated that these output DEGs might participate in a transition to a second night of stress that seems to be a relevant time for Brachypodium survival under submergence (Figure 1B).

Enriched GO terms by down-regulated DEGs were mostly related to photosynthesis processes such as carbon fixation, plastid, chlorophyll metabolism, stomata closure, and starch metabolism (Figure 2C; Table S2). These processes are known outputs of the circadian clock with a dynamic signaling input from light, energy status, and stress [25]. This highlights that an important result of a changing transcriptome during the day under submergence is contributing to limiting and regulating these known rhythmic and photosynthesis-associated pathways. For example, in Figure 2J, the very abundant transcript *LIGHT HARVESTING PROTEIN B* (*LHB1B2*) started the stress day with reduced expression, had a change in zenith to ZT16, and started already suppressed the next day. The number of GO terms enriched by down-regulated DEGs increased throughout the day (Figure 2D), predicting that most would be gradually repressed as the submergence stress persisted. This transcriptomic response suggests that the result of starting the next day with already down-regulated photosynthesis-related processes is of a predictive nature as it serves both for a day under continued submergence (saving energy) or a day of de-submergence stress (minimizing an oxidative stress related to light exposure).

The GO term enrichment indicated that the transcriptome was dynamically changing in response to time of day and revealed crucial time points of adjustment. However, the appearance of an enriched GO term sometimes depended on the extra presence of only one or two DEGs (specially in Bd21), indicating the need for an additional approach such as individual clustering.

### 2.4. Submergence Stress Reshaped the Expression of Circadian Clock Genes

**Figure 3:**
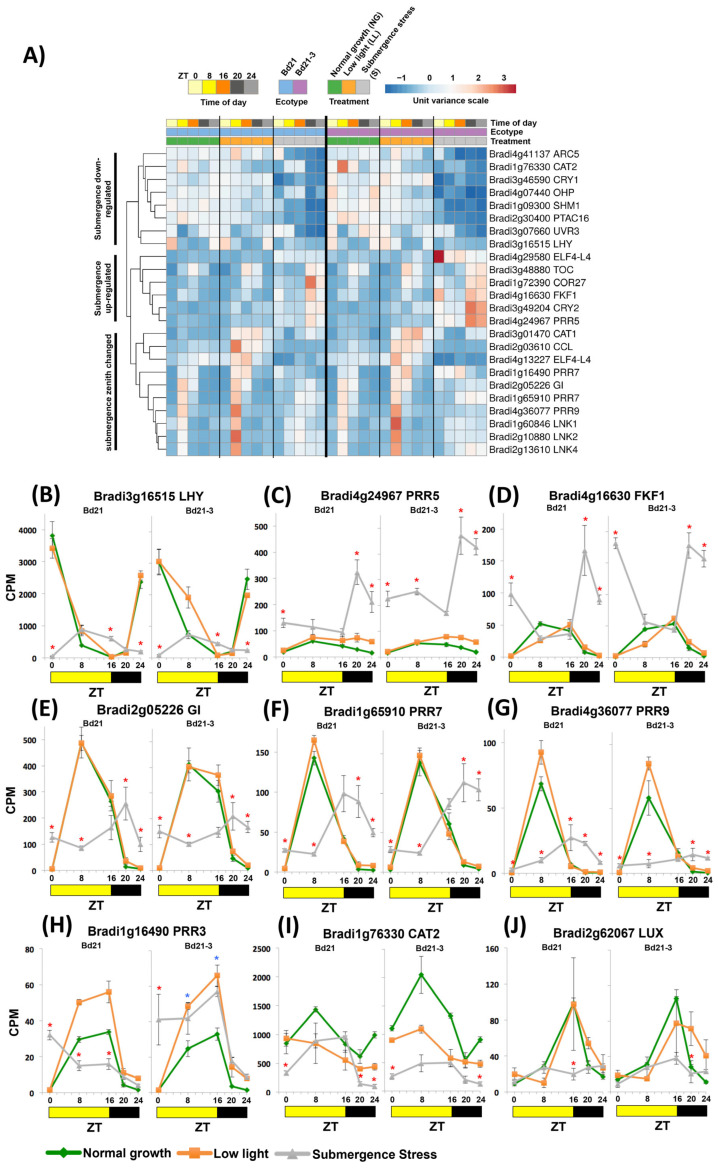
Detection of Brachypodium DEGs of the circadian clock or rhythmic regulation under submergence stress. (**A**) Heatmap of up- and down-regulated DEGs associated with GO terms “circadian rhythm” or “rhythmic processes” clustered. (**B**–**J**) Diurnal expression of selected DEGs associated with the circadian clock under submergence stress, normal growth, and low light controls. Data are means of CPM measured in 3 independent RNA sequencing experiments and error bars indicate SD. Red asterisks indicate significant differences (FDR < 5 × 10^−6^) between submergence stress and low light values; blue asterisks indicate significant differences (FDR < 5 × 10^−6^) between submergence stress values of Bd21 and Bd21-3.

We started by dissecting the GO terms indicating circadian rhythm and rhythmic process; they were enriched in different ZTs both with up- and down-regulated DEGs (yellow arrows in Figure 2C). We clustered all DEGs enriched in these terms and observed separation in three groups (Figure 3A). The first group has down-regulated transcripts, exemplified by the core clock gene *LONG ELONGATED HYPOCOTYL* (*LHY*) with its characteristic expression peak at dawn significantly abated in both ecotypes when compared to LL and NG controls (Figure 3B). The second group contained up-regulated DEGs, such as the midday oscillator component *PSEUDO-RESPONSE REGULATOR 5* (*PRR5*) that showed a dramatic zenith change of 12 h (Figure 3C). The third group included DEGs that showed a change in peak expression towards the middle of the night under submergence. This group included the diurnal clock inductors *NIGHT LIGHT-INDUCIBLE AND CLOCK-REGULATED1* (*LNK 1*,*2*,*4*; Figure 3A), the evening oscillator *GIGANTEA* (*GI*; Figure 3E), and morning oscillator components *PRR7* and *PRR9* (Figure 3F,G). 

Evening Complex (EC) genes such as *EARLY FLOWERING 4* (*ELF4*) had members up- and down-regulated, and *LUX ARRHYTHMO* (*LUX*, Bradi2g62067), despite not being detected in the circadian GO terms, was significantly down-regulated (Figure 3J). The only member of the EC that was not disturbed during the experiment was ELF3 (Bradi2g14290).

We did not find the core evening clock gene TIMING OF CAB EXPRESSION 1 (TOC) as a DEG, because it did not reach the 1.5 Log_2_FC cutoff value (Log_2_FC = 1.44 for Bd21); however, it did show a change in zenith expression from ZT16 to ZT20 when manually included in the clustering (Figure 3A). 

In addition to these core clock genes, other DEGs enriching the diurnal/circadian GO terms consisted of interesting known clock output transcripts. They included oxidative stress enzymes such as *CATALASE 1* and *2* (*CAT1*, *2*) that showed peak transcript abundance in the evening rather than during daytime (Figure 3I), light receptors acting on cell expansion *FLAVIN-BINDING*, *KELCH REPEAT AND F-BOX 1* (*FKF1*; Figure 3D) and *CRYPTOCHROME 2* (*CRY2*) with fully changed zeniths, and the chloroplast coordinator of photosynthetic gene expression *PLASTID-ENCODED RNA POLYMERASE ASSOCIATED PROTEIN* (*pTAC16*) with down-regulated expression (Figure 3A). 

When comparing the stress expression of these DEGs between ecotypes, only *Bradi1g16490* was more up-regulated in the tolerant ecotype (ZT8 and ZT16; Figure 3H). 

This gene seems to be misannotated in the genome as *PRR7* because Higgins et al. [26] found it would be better classified as the *PRR3* ortholog, a gene whose protein is an important post-translational stabilizer of TOC1 protein in Arabidopsis [27]. 

### 2.5. DEGS under Submergence Had ZT-Dependent Transcriptomic Responses

**Figure 4:**
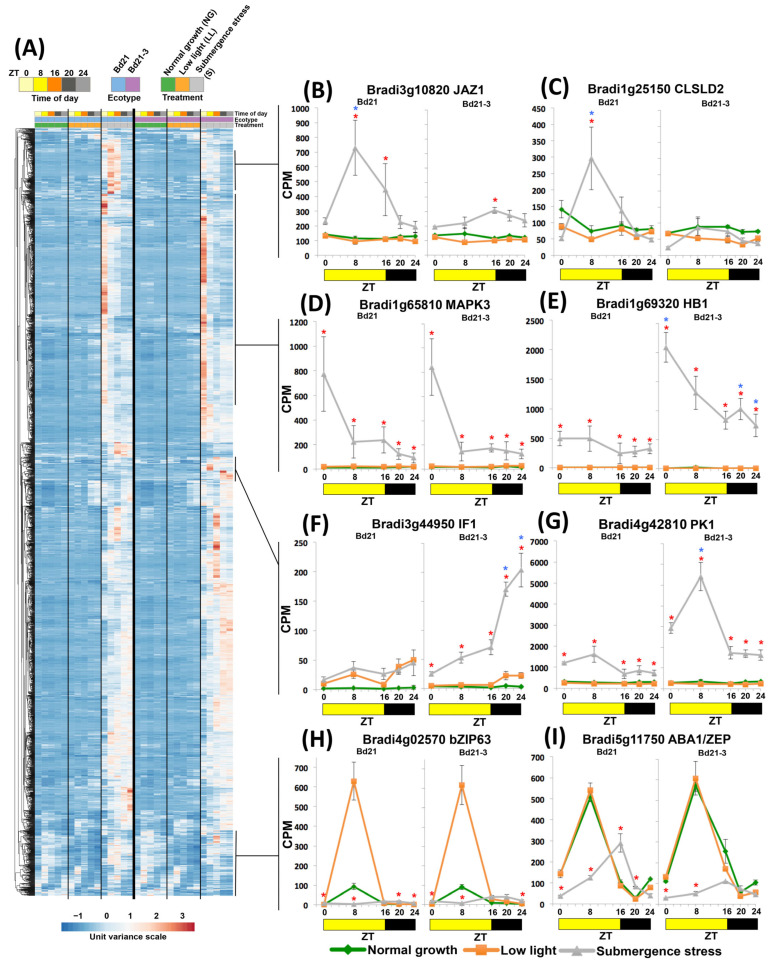
Clustering of up-regulated DEGs in Brachypodium ecotypes Bd21 and Bd21-3 under submergence stress. (**A**) Heatmap of all up-regulated DEGs. (**B**–**I**) Diurnal expression of selected up-regulated DEGs. Data are means of CPM measured in 3 independent RNA sequencing experiments and error bars indicate SD. Red asterisks indicate significant differences (FDR < 5 × 10^−6^) between submergence stress and low light values; blue asterisks indicate significant differences (FDR < 5 × 10^−6^) between submergence stress values of Bd21 and Bd21-3.

**Figure 5:**
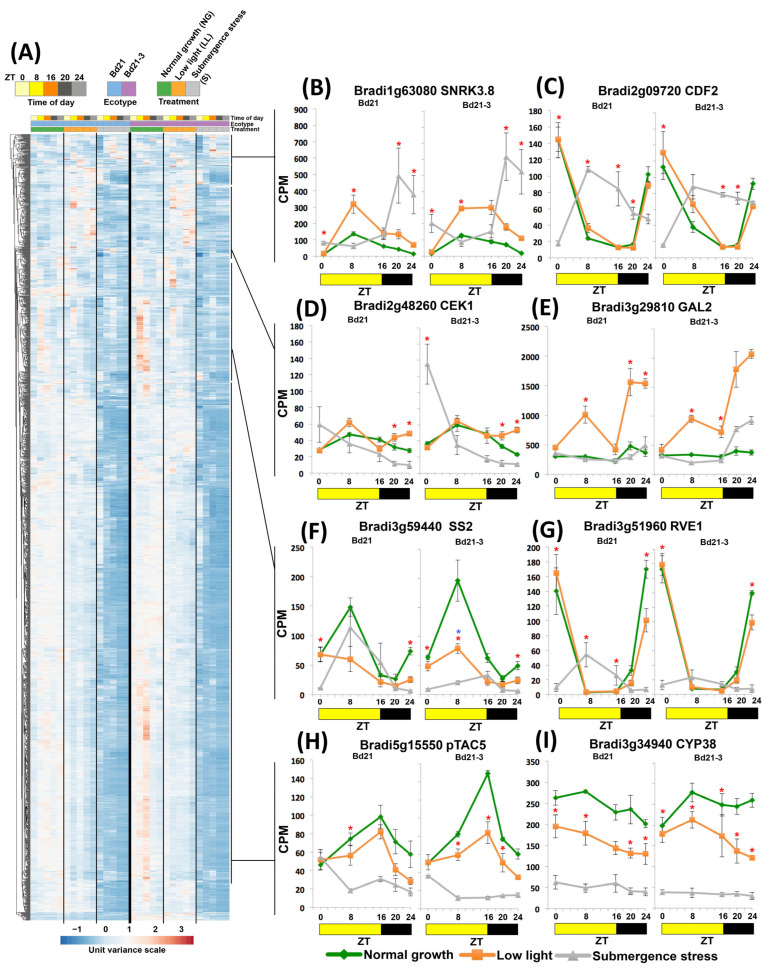
Clustering of down-regulated DEGs in Brachypodium ecotypes Bd21 and Bd21-3 under submergence stress. (**A**) Heatmap of all down-regulated DEGs. (**B**–**I**) Diurnal expression of selected down-regulated DEGs. Data are means of CPM measured in 3 independent RNA sequencing experiments and error bars indicate SD. Red asterisks indicate significant differences (FDR < 5 × 10^−6^) between submergence stress and low light values; blue asterisks indicate significant differences (FDR < 5 × 10^−6^) between submergence stress values of Bd21 and Bd21-3.

We next moved to analyze clusters of up- and down-regulated transcripts (Figure 4 and Figure 5; Table S3). Clustering the up-regulated genes of both ecotypes (2322 DEGs) indicated that 31% grouped as ZT0 (Figure 4A). We hypothesize that this cluster represents DEGs that can be considered early responders during the first night of stress that acquire a more modulated transcriptional response during the illuminated hours. Some were transcriptionally up-regulated in the same intensity by submergence in both ecotypes, exemplified by the signal transducer *MITOGEN ACTIVATED PROTEIN KINASE 3* (*MAPK3*; Figure 4D). Others were more abundant in one ecotype, despite being up-regulated in both; for example, *HEMOGLOBIN 1* (*HB1*) was more expressed in tolerant Bd21-3 (Figure 4E). 

We found clusters that had DEGs with complex patterns of altered diurnal expression under submergence. For example, *JASMONATE-ZIM-DOMAIN PROTEIN 1* (*JAZ1*) and *CELLULOSE SYNTHASE* (*CLSLD2*) acquired an expression zenith at ZT8 that was not present under LL or NG controls, and interestingly, were more up-regulated in sensitive Bd21 (Figure 4B,C). Other patterns were observed with transcripts being up-regulated by LL but shifting its zenith to the night under submergence (e.g., *bZIP63*; Figure 4H) or shifted their peak transcript accumulation to the night but were not altered by LL (e.g., *ABA1/ZEP*; Figure 4I).

An interesting cluster of DEGs contained genes that showed significantly higher transcript accumulation throughout the day in tolerant Bd21-3. We highlight *Bradi3g44950* coding for a *MITOCHONDRIAL F1F0-ATPASE INHIBITOR FACTOR 1* (*IF1*) that accumulates in the night (Figure 4F) and *PYRUVATE KINASE* (*PK1*; Figure 4G) more expressed in the illuminated period. 

When analyzing down-regulated clusters, some DEGs acquired a new zenith. Examples include a member of the sugar transducers family, *SUC-NONFERMENTING1-RELATED PROTEIN KINASE 3.8* (*SnRK3.8*, [28]; Figure 5B), which shifted its peak transcript accumulation from midday to the night, or the flowering inhibitor and miRNA regulator *CYCLING DOF FACTOR 2* (*CDF2*), which inverted its known rhythmic diurnal pattern (Figure 5C; Diurnal database [29]). There were down-regulated DEGs that had a steady descending pattern exemplified by the endoplasmic reticulum-protective enzyme *CHOLINE/ETHANOLAMINE KINASE 1* (*CEK1*; Figure 5D). Another group of genes was strongly induced by light but not by submergence, and thus classified as down-regulated DEGs, such as the leaf expansion enzyme *ALPHA-GALACOTSIDASE* (*GAL2*, [30]; Figure 5E). A third gene cluster consisted of DEGs losing their zenith peaks, e.g., *STARCH SYNTHASE2* (*SS2*) that showed a reduced midday zenith peak in tolerant Bd21-3 (Figure 5F), or the clock-regulated transcription factor *REVEILLE 1* (*RVE1*) that had a zenith shift from dawn to midday in sensitive Bd21 or strong down-regulation in tolerant Bd21-3 (Figure 5G).

As predicted by accumulated GO terms, the largest cluster (1139 DEGs, 53%) belonged to genes coming out of the night with LL expression levels and eventually being down-regulated under submergence, or coming out from the night with already down-regulated levels. Examples of the above are the chloroplast coordinator and retrograde signal *pTAC5* (Figure 5H) and the chloroplast protein-folding enzyme *CYCLOPHILIN 38* (*CYP38*, Figure 5I), both known diurnal rhythmic genes (Diurnal database [29]). As observed in Figure 5A, most of these genes were diurnally regulated in normal growth conditions, indicating that an important output of the altered expression of the clock genes may be this multigene down-regulation and loss of diurnal expression.

### 2.6. Ecotypes of Contrasting Tolerance Have Exclusive DEGs with Acquired ZT-Dependent Expression

**Figure 6:**
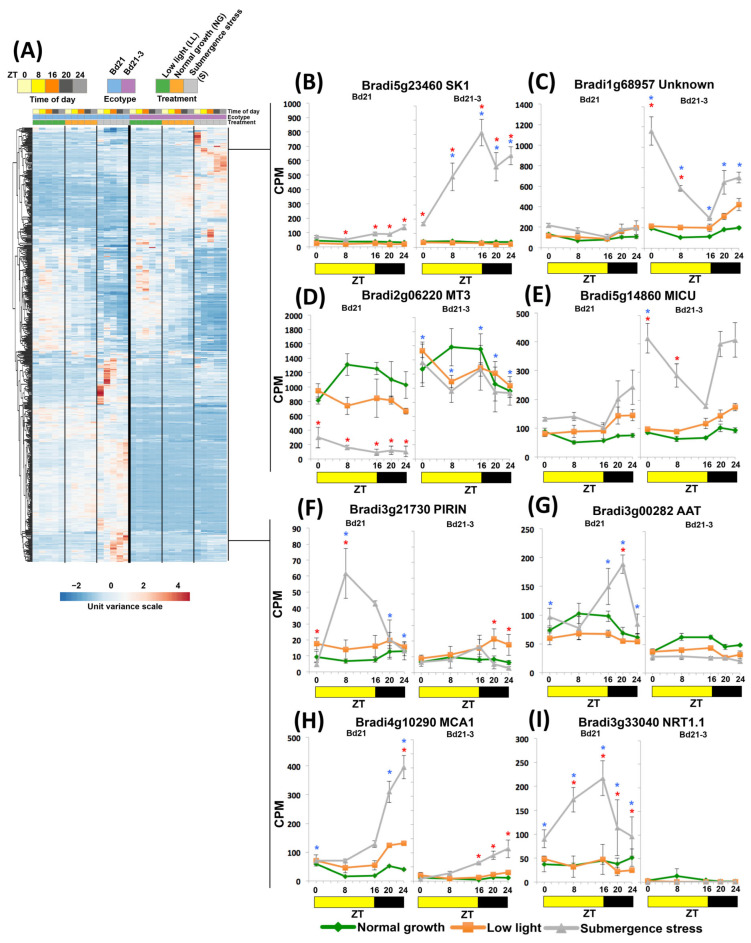
Clustering of DEGs when the submergence stress transcriptomes of Brachypodium ecotypes Bd21 and Bd21-3 are compared. (**A**) Heatmap of all DEGs Bd21S/Bd21-3S. (**B**–**I**) Diurnal expression of selected DEGs preferentially expressed in either Bd21 or Bd21-3; data are means of CPM measured in 3 independent RNA sequencing experiments and error bars indicate SD, red asterisks indicate significant differences (FDR < 5 × 10^−6^) between submergence stress and low light values, blue asterisks indicate significant differences (FDR < 5 × 10^−6^) between submergence stress values of Bd21 and Bd21-3.

We highlighted in the previous sections some interesting differential transcripts found in the context of GO or clustering, for example, *PAL*, *AOX1*, *PRR3*, *JAZ1*, *SS2*, *CLSLD2*, *HB1*, *PK1*, *IFI1*, and *ZEP*. Because of these observations, we directly contrasted both ecotypes by comparing their expression values only in the submergence conditions and excluding LL controls. In this manner, we found 526 DEGs that were contrastingly up- or down-regulated at any ZT on the ecotypes studied (Figure 6A; Table S4). Clustering these genes showed a complex scenario of increased or reduced zenith peaks and changes in amplitude (transcript abundance).

In the tolerant Bd21-3, for example, the abundance of the enzyme-coding *SHIKIMATE KINASE 1* (*SK1*) transcript accumulated steadily at a higher rate (Figure 6B), while *Bradi1g68957* (a *DEG* with unknown identity; Figure 6C) and *MITOCHONDRIAL Ca^2+^ UNIPORTER COMPLEX* (*MICU*) acquired higher amplitudes with peaks at dawn (Figure 6E). Others remained expressed statistically steady in tolerant Bd21-3 but changed in sensitive Bd21. We highlight the cysteine-rich *METALLOTHIONEIN 3* (*MT3*) that was strongly down-regulated in Bd21 under submergence (Figure 6D). In this category, *MT3* was the most abundant differentially expressed transcript in Bd21-3 together with *Bradi2g62315*, another *MT*. 

In contrast, DEGs such as a PIRIN-like lignin accumulation suppressor (*PIRIN*), a *TRANSMEMBRANE AMINO ACID TRANSPORTER* (*AAT*), and a *NITRATE TRANSMEMBRANE TRANSPORTER* (*NRT1.1*) showed peak transcript accumulation at specific ZTs only in sensitive Bd21 while remaining unresponsive in submerged BD21-3 (Figure 6F,G,I). Finally, another group showed increased transcript accumulation in sensitive Bd21 relative to Bd21-3; an example is *Ca^2+^-PERMEABLE MECHANOSENSITIVE CHANNEL* (*MCA1*; Figure 6H). 

### 2.7. Arabidopsis Clock Genes Expression Is also Reshaped under Submergence Stress

**Figure 7:**
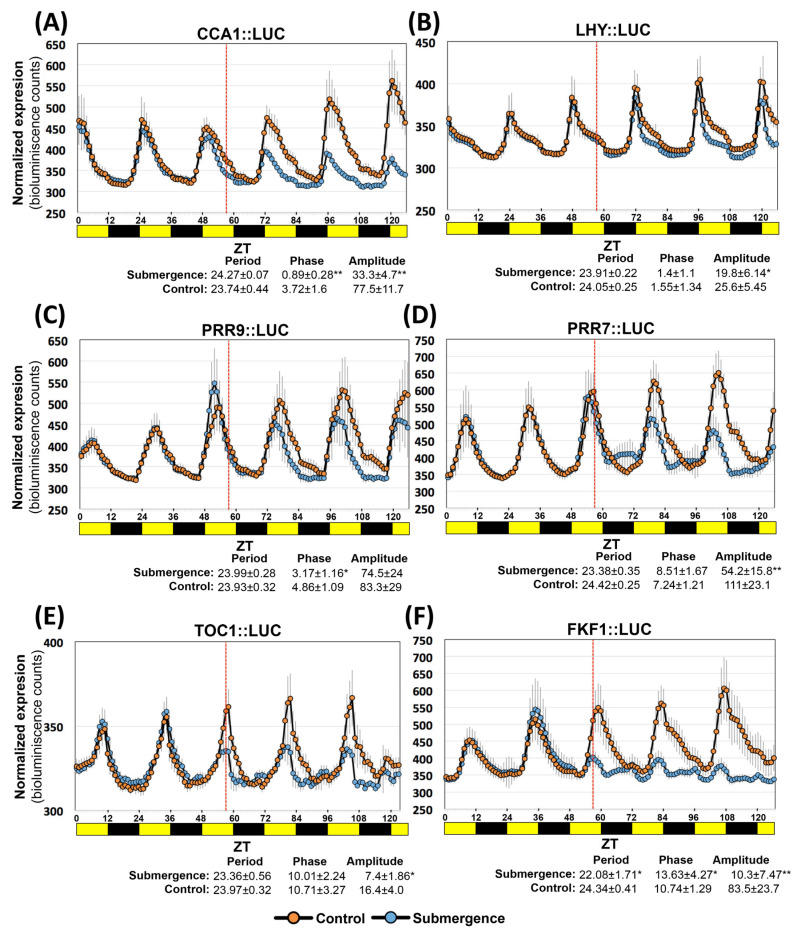
Diurnal expression of Arabidopsis circadian clock genes under submergence stress. (**A**–**F**) Bioluminescence measurements of seedlings expressing the indicated promoter luciferase constructs. Seedlings were grown in 12 h light/12 h dark cycles and constant temperature for 7 d before imaging. The red line indicates the time (ZT56) when the seedlings were submerged. Orange lines and blue lines represent average values of control and submergence seedlings, respectively. Yellow and black boxes indicate light and night, respectively. Data in graphs are means of 3 independent experiments (*n* = 12–14 seedlings); error bars indicate standard deviation (SD). For period, phase, and amplitude, data are means of the same experiments with standard deviation. Asterisks indicate significant differences (* *p* < 0.01, ** *p* < 0.001) indicated by one-way ANOVA with post hoc Tukey HSD.

To investigate if the Arabidopsis circadian clock genes respond similarly as Brachypodium during submergence, we monitored bioluminescence changes in Arabidopsis *CCA1*, *LHY*, *PRR9*, *PRR7*, *TOC1*, and *FKF1* promoter luciferase seedlings. One difference between the clock genes of Brachypodium, rice and Arabidopsis is that monocots only have one CCA1/LHY gene, while Arabidopsis has two components [26]. Interestingly, *CCA1* showed reduced amplitude that started the first day after imposing the stress while LHY was less responsive (Figure 7A,B). For *PRR9*, a shift in the timing of peak expression was observed immediately after the stress (Figure 7C).

*PRR7* in Arabidopsis did not show a reduction in its zenith peak at midday or a shift in peak expression, as observed for Brachypodium. Although strategies for *PRR7* expression in Brachypodium and Arabidopsis were different under submergence, they appear to exhibit a similar pattern of an up-regulated expression of *PRR7* at night and at dawn the first day (Figure 7D). Similar to Brachypodium, *TOC1* was down-regulated in Arabidopsis immediately after stress; however, no change in peak timing was observed (Figure 7E). *FKF1* expression had converse expression in Arabidopsis with respect to Brachypodium. Furthermore, we detected significant changes in phase and/or amplitude for all clock genes tested (Figure 7F). 

The use of reporter lines allowed us to determine that the circadian clock genes are also affected by submergence stress in Arabidopsis. Although the mechanisms of both plants were not mirror strategies, they probably are reflecting age-specific differences, sensibility to carbon/energy depletion, and LHY/CCA1 specialization.

### 2.8. The Hypoxia Core Genes in Brachypodium Are Early Responders

**Figure 8:**
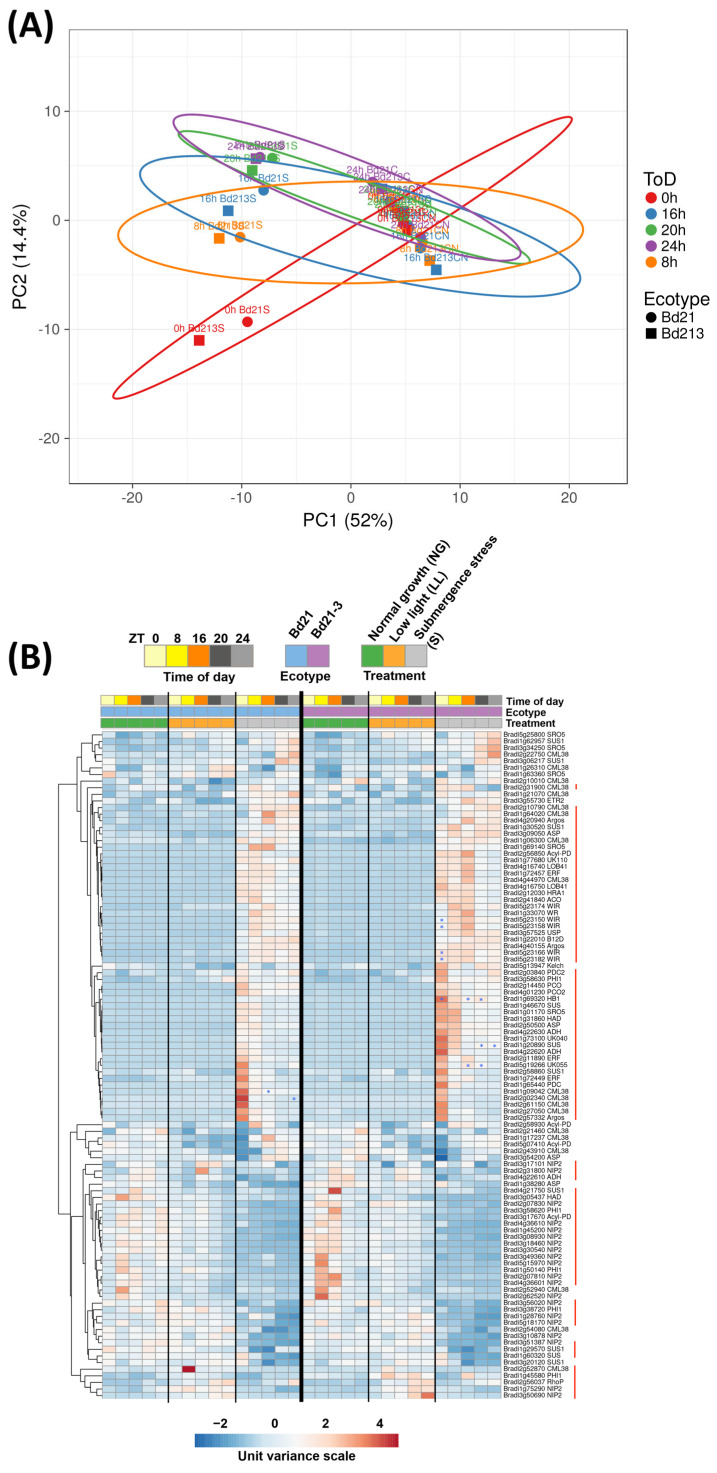
Clustering of Brachypodium homologs to the Hypoxia Core Genes (HCG) from Arabidopsis. (**A**) Principal component analysis where X and Y axis show principal component 1 and principal component 2 that explain 52% and 14.4% of the total variance, respectively. Prediction ellipses are such that with probability 0.95, a new observation from the same group will fall inside the ellipse. (**B**) Heatmap of all HCG homologs (DEGs or not). Only homologues with more than 15 CPMs in at least one column were analyzed. Data are means of CPM measured in 3 independent RNA sequencing experiments. Red lines next to each transcript indicate significant differences (FDR < 5 × 10^−6^) between submergence stress and low light values in at least one point; if no line is included, it indicates that there are no differences in that row. Blue asterisks inside the squares indicate significant differences (FDR < 5 × 10^−6^) between submergence stress values of Bd21 and Bd21-3.

Finally, we took the Hypoxia Core Genes (HCG), as reported by Lee et al. [31], and searched for all annotated homologs in Brachypodium using Phytozome [32] and TAIR [33] to observe their expression in our datasets. A principal component analysis indicated that the expression under submergence stress at ZT0, and to a lesser degree, ZT8, were the times of the day that differentiated that data subset (Figure 8A). Through a homology search, and as shown in Figure 8B, we observed that not all transcripts were significantly up-regulated—some were down-regulated, and others were not significant (Figure 8B). At least 1 homolog of 33 of the original 34 members of the HCG was significantly up-regulated, especially in the ZTs revealed by PCA. Their presence with high expression after the first night of stress indicate that they can be considered as early responders. Only the HCG At4g39675 does not have homologs in monocots.

We found that some of those significantly up-regulated HCGs were also more expressed in tolerant Bd21-3. We previously noted *HB1*, and now six more transcripts were found. Four are members of the *WOUND-INDUCED RESPONSE* (*WR/WIR*) family of small polypeptides (also named *WOUND-INDUCED POLYPEPTIDES*; *WIPs* [34]), one a member of the *SUCROSE SYNTHASE* (*SUS*) family and the homologue of *AT1g33055* (*Bradi5g19266*, abbreviated as *UK055*), a gene of unknown function. On the other hand, two members of *CALMODULIN-LIKE 38* (CML-38) were more up-regulated in the sensitive ecotype.

## 3. Discussion

By phasing efforts, integrating signals, and executing predictions based on the species history, the circadian clock components of plants have allowed the evolution from simple and direct responses into versatile adaptations needed to respond in a dynamically changing environment [35]. One of such changing features is the balance of water and energy, an ecological component that greatly shapes plant diversity and architecture across the globe and in small ecological niches [36,37]. 

We propose that excess of water and associated compound stresses are temporary historic costs to pay for adequate availability of water that should be integrated with the circadian clock response of the plant. The strength of the response may depend on the precipitation, soil drainage, and topography that shape plant adaptation. Supporting this idea, research has discovered genetic diversity in plant strategies for survival and adaptation to water excess stresses in rice [38], Arabidopsis [39], maize [40], Brachypodium [12], and Rumex [41]. This diversity has found application, especially in rice, in the development of Sub1-rice varieties tolerant to submergence stress developed on the basis of the discovery of allelic variation on Ethylene-Responsive Factors (ERFs) [42].

In this work, we aimed to expand our knowledge on the molecular dynamics of submergence stress in Brachypodium—first, to know if the submergence stress response changes throughout the day; then, to observe if the response of DEGs with gene ontology related to rhythmic expression or components of the circadian clock and its outputs changed in the context of submergence stress; and finally, to learn which DEGs are differentially expressed by ecotypes of contrasting tolerance. The experimental approach was to compare the transcriptomes expressed throughout a diurnal cycle. For that purpose, ecotypes of the model grass plant Brachypodium with contrasting tolerance to submergence stress were analyzed. 

### 3.1. The Transcriptomic Response of Brachypodium Is Dynamically Responding to Time of Day

First, we compared the fitness of two previously studied ecotypes (Bd21, Bd2-3) and a newly included ecotype (Bd21-3) under submergence stress applied with a long-day diurnal cycle. Since submergence limited the light reaching the plant, our main comparison control was low-light-irradiated plants growing in parallel (~45% less irradiance), but we also kept normal growth controls. As previously reported [12], Brachypodium ecotypes displayed a quiescent response and leave damage proportional to the time of submergence (Figure 1). However, Bd21-3 survived longer under submergence, maintained more green leaves after the stress, and could recover better than the sensitive Bd21 and intermediate Bd2-3 ecotypes when using damage and number of leaves as fitness indicators. Therefore, Bd21-3 and Bd21 should have molecular components expressed differentially that could explain the distinction in fitness.

We opted for a transcriptomic approach to compare the differential expression of the contrasting ecotypes and explore the connection of submergence stress with the diurnal cycle. We decided to characterize the expression in the first day of stress (Figure 1D), reasoning that in both ecotypes, this is a non-lethal stage (Figure 1 and Figure S1), so the transcriptome that we would measure is the base of the observed differential fitness at that level of genetic information. We chose this experimental point expecting that the transcriptomes would not reflect a plant starting to set its response or a characterization of a lethal period of stress. We considered that this was achieved because the quantification of DEGs at different ZT indicated that the transcriptome was changing dynamically in a complex manner with dozens of genes regulated at specific times of the day (Figure 2A,B).

In this way, we found that the largest ecotype-shared and individual group at ZT0 was enriched with kinases, either acting as metabolic enzymes (such as PFK3 and PK1, Figure 2E and Figure 4G, respectively) or signaling pathways (MAPK3, Figure 4D). Remarkably, it contained many uncharacterized genes. For example, up-regulated DEGs in tolerant Bd21-3 at ZT0 contained 59% of unannotated genes and 15% of completely unknown function (*n* = 168). It can be proposed that these transcripts are strong early responders after the initial night of stress.

### 3.2. The Expression of Known Circadian Clock Genes and Outputs Is Affected by Submergence, a Multicomponent Stress

As hypothesized, known transcripts of the circadian clock were found to be misregulated at different ZTs under submergence stress in Brachypodium. The global analysis of these components (Figure 3A) suggested that the expression of clock genes was adjusted to general delayed nightly zenith peaks. Low light did not alter the circadian clock expression when compared to normal growth plants (Figure 3), pointing to submergence-related stressors as the Zeitgebers (e.g., [ATP], [NADH], [CO_2_/O_2_]). All components came out of the first night of stress exhibiting altered responses, suggesting that regardless of the identity of the submergence Zeitgebers that induced these changes, the plant may use them in a predictive way to be prepared for the next day either under continuing submergence stress or under de-submergence stress [43].

One essential component of the submergence stress that would act as input for these changes could be carbohydrate status. Free sugars are carefully regulated, because their insufficiency causes an energy stress [44], while their excess causes an osmotic stress [45]. Haydon et al. [46] observed that in Arabidopsis, free carbohydrates advance the circadian clock period and their absence delays it. This is achieved through up-regulation of *PRR7*, a diurnal repressor of the early morning oscillator *CCA1*. Frank et al. [28] expanded the components of this pathway by determining that sugar signaling is partially executed through bZIP63 acting as a transcriptional activator of PRR7. We detected all the components of this signaling module in our submergence transcriptome being expressed similarly to Arabidopsis in a no-sucrose status. *PRR7* and *bZIP63* are up-regulated with a delayed peak, while *LHY* was down-regulated at dawn. (Figure 3B,F and Figure 4H). In Arabidopsis, the expression of *PRR7* and *CCA1* was also altered under submergence (Figure 7). Haydon et al. [46] reported the phenomena of stress gating for carbohydrates acting as Zeitgebers, i.e., when identical stimuli result in different molecular responses depending on the time of day when they are applied. Stress gating of the diurnal and circadian molecular responses has been reported for light [47,48], temperature stress [49,50], drought stress [51], and redox balance [52]. In the future, it will be interesting to determine if imposing submergence stress at different times of the day has a gating effect frame with intensities of output responses different from the one we tested (late evening).

Higgins et al. [26] discussed that the components of the PRR family in Brachypodium may not be comparable to those in Arabidopsis in a straightforward manner. For example, an interesting difference is the fact that Brachypodium and rice only have one *CCA1*/*LHY* gene, while Arabidopsis has two components. It has been reported that although CCA1 and LHY have overlapping functions in Arabidopsis, they have hundreds of separate output target genes (one-third in common), e.g., ABA biosynthetic genes being targeted only by LHY [53]. The observations reported here support the involvement of genetic components of the clock for Brachypodium and Arabidopsis under submergence stress. 

### 3.3. DEGs Present a Picture of Complex Interplay under Submergence between Metabolism, Signaling and Rhythmic Processes

The analysis of the outputs of these submergence-altered clock components suggests that the expression of circadian clock genes under submergence is evolutionary helpful and not only symptomatic. Starting with the clock components themselves, some have known direct effects that may help plant survival. This is the case of PRR3, a reported post-translational stabilizer of TOC1 [27], which was the only differentially up-regulated clock gene in tolerant B21-3 during the day (Figure 3H). We propose that this would be the basis of the high diversity of GO processes detected at ZT16 in tolerant Bd21-3 since TOC1 not only is a master repressor of the oscillating clock components but also a transcription regulator that influences dozens of diurnal processes [54] and, when misregulated, changes the metabolome and the ATP/ADP ratio, with severe consequences for plant architecture [23]. Another interesting gene is *FKF1*, a repressor of cellulose synthesis and elongation [55]; its strong up-regulation can indicate that it is a crucial component of the quiescent response under submergence (Figure 4D). Finally, *RVE1*, a clock component that promotes enzymes for starch degradation and glucose catabolism [56], is more down-regulated in tolerant Bd21-3 and may be associated with the up-regulation of starch metabolism transcripts in sensitive Bd21.

Outputs outside of the clock components were diverse and complex. Excluding the ZT0 up-regulated DEGs discussed above as early responders, 75% of up-regulated and most down-regulated genes had transcription differences according to the ZT analyzed, indicating the long-reaching consequences of clock reconfiguration in the submergence response. We found well-known responses to submergence stress in up-regulated DEGs being adjusted on their canonical diurnal oscillation such as starch synthesis, amino acid catabolism, hormone biosynthesis, and pyruvate metabolism. 

For down-regulated DEGs, the photosynthesis process was a predominant aspect of DEGs exhibiting reduced amplitude. We highlight retrograde signals involved in chloroplast biogenesis such as pTACs [57], secA (*Bradi2g12067*; [58]), and CRY2, the latter by not changing its zenith but increasing its transcript abundance, and may be a trigger to restart photosynthesis recovery and probably clock resetting [25,57].

We emphasize for future studies calcium signaling, a physiological aspect involving DEGs that responded with new zenith peaks under submergence. This is exemplified by MICU, a channel regulator that severely impacts mitochondria microstructure and changes substrate preferences of the electron transport chain in Arabidopsis [59]. Interestingly, we found *MICU* both as a ZT0 early responder and as a DEG acquiring zenith expression at dawn only in tolerant Bd21-3 (Figure 6E). Conversely, *MCA1*, a Ca^2+^ channel [60], was up-regulated as a late responder more in Bd21 (Figure 6H).

Some mechanisms that have also been previously described in tolerant plants [12,40,41,61] were detected. We observed augmented ROS-related transcripts in the sensitive ecotype, such as *HSP* and *AOX1* (Figure 2G,I), as well as starch synthesis and degradation (Figure 5F). Likewise, components of the nitric oxide cycle, such as *HB1* up-regulation, were confirmed in tolerant plants and expanded to include nitrate transport, exemplified by *AAT* and *NRT1.1* up-regulated in sensitive Bd21 (Figure 6G,I). Glycolytic transcripts, aromatic acid pathways, and lignin modulation were also enriched. As expected, Brachypodium has up-regulated DEGs homologues to 33 of 34 components of the Hypoxia Core Genes (HCG) as defined in Arabidopsis [31] and do not seem to have a rhythmic diurnal expression, indicating that they are early responders (Figure 8). Seven HCGs were differentially up-regulated in the tolerant ecotype: the above-mentioned *HB1*, four members of wound-responsive polypeptides, one SUS member, and an uncharacterized transcript coding for a short peptide. 

The direct comparison of submergence transcriptomes between the two ecotypes used allowed for the discovery of molecular dynamics that may contribute to our knowledge of differential tolerance mechanisms. We underline an interesting transcript coding for *IF1*, a small protein that has received much attention in mammal systems as a crucial control point for ATP production by mitochondrial ATP synthase and as an enhancer of glycolysis with roles in cancer and metabolic diseases [62]. In contrast, IF1 has begun to be characterized in plants and has shown similar features to its mammalian counterparts, such as mitochondrial localization, an increase in the ATP/ADP ratio, and the suppression of ROS stress markers [63,64]. The expression pattern of *IF1* may indicate the entrance of the plant to a new stress phase in the second night where the energy stress may be more imperative; the sensitivity of this gene to cell energy status is an interesting hypothesis to follow. The finding of *IF1* up-regulated in the tolerant ecotype under submergence stress is logical in the context of energy management and is promising for further biotechnological characterization.

In addition to IF1, we point out as interesting mechanisms the most abundant DEGs in Bd21-3, *Bradi1g68957*, an unknown function transcript with acquired strong early-morning expressions under stress, and the multirole *MT3*, with structural capacities of buffering ionic metals and controlling oxidative stress simultaneously [65].

**Figure 9:**
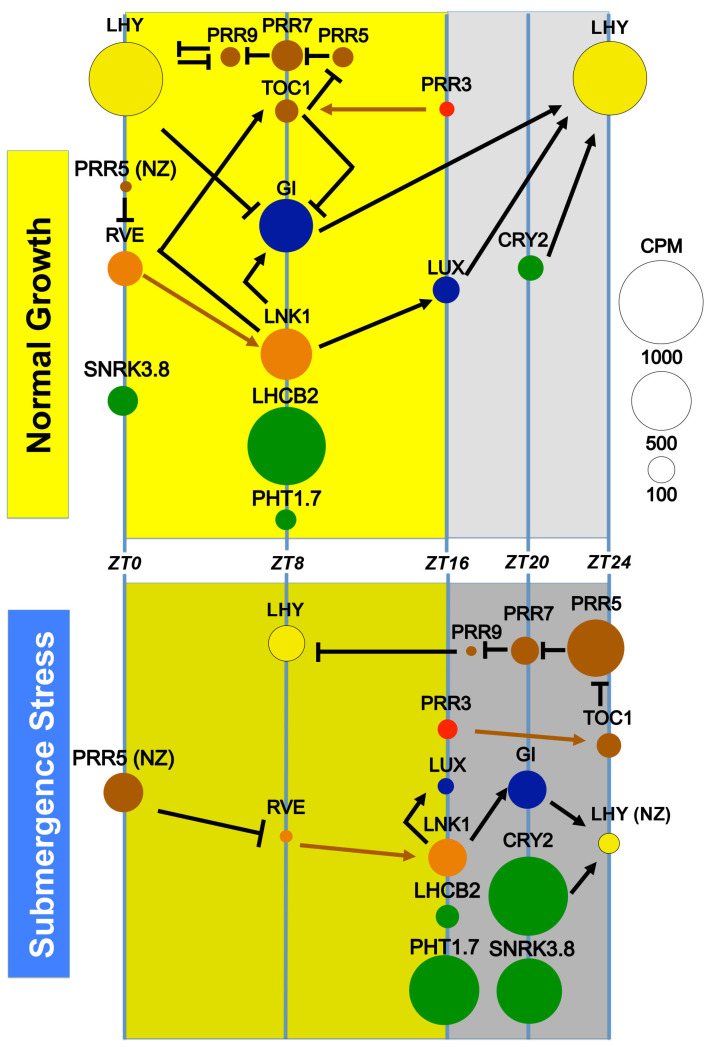
Model of the altered relations between the clock genes induced by submergence stress in Brachypodium observed in this work. Normal growth plants have a standard clock gene expression allowing the coordination of outputs in a manner that optimizes the energy status with the available resources on an unstressed day. Under submergence stress, core gene clocks shift their zenith peaks to the night, creating a new clock configuration that creates new diurnal oscillation patterns and/or repression of the previous patterns to achieve a maximal energy status with the scarce available resources under stress. We exemplify the new configuration of outputs with SNRK3.8 and PHT1;7 up-regulated and phase-shifted transcripts; LHCB2, a down-regulated and phase-shifted transcript; and CRY2, an up-regulated transcript without phase shift that may itself affect the clock. Circled areas represent the measured CPM for each transcript, except LHY and LHCB2, which are down-scaled to 50% and 25% of their area to fit, respectively. The position of the circle indicates the zenith peak detected for each gene, except when non-zenith (NZ) is indicated. Black lines indicate transcriptional relationships and brown lines indicate protein interactions (as condensed for Arabidopsis [25]). Circle colors indicate early morning genes (yellow), midday genes (brown), morning responsive genes (orange), evening genes (blue), protector of TOC1 (red), and output genes (green).

With these observations, we present a model summarizing the response of the clock genes during submergence stress in Brachypodium based on connections defined in Arabidopsis (Figure 9). During normal growth, the circadian clock components are expressed in their known standard periods and zenith peaks to coordinate a set of outputs useful for such optimized conditions. We exemplify SNRK3.8 (a sugar signaling component), LHCB2 (a photosynthetic antenna), PHT1.7 (a phosphorus transporter), and CRY2 (a photoreceptor and clock rhythm marker). When the submergence stress is sensed, most likely by multiple simultaneous reporter molecules (e.g., NO/O_2_/CO_2_, ethylene, sugars, amino acids), the clock components rapidly alter their expression, shifting to nightly zeniths of expression. We speculate that this has long-reaching effects in down-regulating the amplitude of photosynthesis and chloroplast transcripts (e.g., *LHCB2*), repositioning energy-demanding activities such as P/N transport (e.g., *PHT1.7*), modifying zenith peaks for signaling cascades (e.g., *SNRK3.8*), and up-regulating the expression of sensing mechanisms for the predicted retreat of submergence and adjustment of clock and output gene expression (*CRY2*). Finally, the clock components themselves (e.g., CCA/LHY, TOC1), through their down-regulation, can release the repression of their transcriptomic targets with roles in plant metabolism signal energy depletion (e.g., *PRR7*) and relocating their transcriptomic activity for further establishment of the modified clock (*PRR5*).

It has been proposed that the circadian clock not only allows the response to signals and optimization of gating and phases, but also gives context to the evolutionary and recent history of the plant [35]. These dramatic changes observed in the time of day expression of clock transcripts and its primary outputs in response to submergence stress suggest that this diurnal expression is coordinated and may be historically relevant.

### 3.4. Conclusions

We reported the transcriptomes of two Brachypodium ecotypes of differential tolerance to submergence throughout a day of stress in a non-lethal period. This allowed us to observe that the transcriptome changed dynamically according to the time of day and that these new transcriptional responses included an adjustment of gene expression for several circadian clock components. By characterizing Arabidopsis clock gene promoters under submergence, we propose that adjusted clock gene regulation can be considered as a standard plant response to submergence stress. The alteration in response to stress in Brachypodium included hundreds of components showing reduced diurnal expression, acquiring new zeniths, or changing their amplitude of expression. The dataset presented and discussed here can be used to explore the fitness advantages acquired through the evolution of this response, the role and use of the differential tolerance markers and its promoters in the field of biotechnological chronoculture, and for expanding our knowledge of submergence stress.

## 4. Materials and Methods

### 4.1. Brachypodium Germplasm 

Seeds of Brachypodium ecotypes Bd21 and Bd2-3 were propagated from original seed obtained from Professor David Garvin (USDA). Bd21-3 seeds were obtained from the Joint Genome Institute (DOE).

Seeds were surface disinfected with 1:1 household bleach (sodium hypochlorite 1.6%) in distilled, deionized, and autoclaved water (ddH20), rinsed five times in 20 mL of ddH20, and scarified in water for 4 d at 4 °C. Seeds were sown horizontally in substrate (1:4 *v*/*v* perlite:Cosmopeat) previously autoclaved for 2 h, cooled down, and mixed with 2% *w*/*w* slow liberation fertilizer (NPK 15:15:17 Nitrofoska, EuroChem, Mexico). Germination and growth were under long-day conditions (16 h light/8 h dark, 150 μE m^−2^ s^−1^, 50% humidity) in a growth room, with irrigation every 3 d using filtered tap water. All pots had small stones at the bottom (~15% of pot volume) to avoid buoyancy. 

### 4.2. Submergence Stress in Brachypodium

Brachypodium plants (14 days old after germination, 6-leaf stage) were randomly arranged and submerged (S samples) in filtered tap water (30 cm deep) inside opaque-walled plastic tanks filled 24 h before the experiment. Plants collected at each indicated time were always grown in the same tank. Light reached the plants subjected to submergence stress (S) at 70 μE m^−2^ s^−1^. Normal growth controls (NG) were left at 150 μE m^−2^ s^−1^, while low light controls (LL, 70 μE m^−2^ s^−1^) were moved to plastic tanks without submergence next to submergence tanks until the end of the experiment (6 d).

As indicated in Figure 1D, submergence stress started at ZT13 (3 h before night) and was stopped at the indicated times by the gentle subtraction of pots from the water column and immediately put to grow under NG conditions. We registered the number of leaves and classified them by appearance in turgent (full green), chlorotic (partial or total yellowish), or senescent (full brownish). Boxplots were built using BoxPlotR [66].

### 4.3. RNA Sequencing

Brachypodium ecotypes Bd21 and Bd21-3 were subjected to submergence stress as previously described and collected as detailed in Figure 1D. Aboveground tissue was collected, frozen immediately in liquid nitrogen, and stored at −80 °C. Tissue was ground to powder with a mortar and pestle with liquid nitrogen, avoiding thawing. Samples of S, NG, and LL controls were collected in independent triplicates at the indicated points, and each replicate consisted of a pool of 5 plants. Total RNA was extracted with the kit Direct-zol RNA mini prep kit (Zymo Research, R2050, USA) and digested in-column with provided DNAse I. Plants were grown in parallel but not collected to verify the stress intensity in the collected plants (Figure S1).

RNA integrity and concentration were verified in formaldehyde-denaturing 1.0% agarose gels, Nanodrop 2000 (Thermo Scientific, USA), and in a Bioanalyzer 2100 (Agilent, USA). Samples with an RNA Integrity Number (RIN) between 6.4 and 7.2 were used for indexed-library construction (90 libraries) and sequencing in an Illumina NextSeq 1x100 format with up to 15–20 million reads per library. To ensure adequate reads from each library, some libraries were repeatedly sequenced producing more than one FASTQ file. RNA sequencing was performed as a service at the Laboratorio Nacional de Genómica del CINVESTAV-IPN; http://www.langebio.cinvestav.mx/labsergen/, (accessed on 1 March 2023). 

### 4.4. Bioinformatic Analysis

A total of 149 RNA sequencing FASTQ files were mapped to the *B. distachyon* genome (Bdistachyon_314_v3.0.fa.gz: JGI v.3.0 assembly; [32]) using HISAT2 (v.2.1.0; [67] to generate Binary Alignment Map (BAM) files. For libraries with more than one FASTQ file, the resulting BAM files were combined using SAMTools (v.1.12; [68]). Libraries that failed to produce 90% read alignment were discarded (Table S6). BAM files were subjected to read count by HTseq python library (v.1.0; [69]). Gene expression and count per million (CPM) values were calculated using the edgeR program [70], with the GLM (generalized linear model) method using an FDR cutoff of <0.05. RNA sequencing data were deposited at NCBI GEO (GSE215159) and are given analyzed in Table S5.

Transcripts were considered as differentially expressed genes (DEGs) when the logarithmic fold change of count per million (Log_2_FC[CPM]) value was ≥ 1.5 (up-regulated) or ≤ −1.5 (down-regulated), concomitant with an FDR < 5 × 10^−6^, and at least an average of 15 counts per million (CPMs). DEGs were separated by VennDiagram, https://bioinformatics.psb.ugent.be/webtools/Venn/ (accessed on 13 August 2022). GO analyses and graphical representations were performed using PANTHER [71] and REVIGO [72] as described [73]. DEGs were clustered using ClustVis [74] by Euclidean distance and average linkage of CPM values scaled to unit variance, where a difference of 1 means that the values are one standard deviation away from each other in that row.

### 4.5. Bioluminescence and Submergence Stress in Arabidopsis

Clock promoter luciferase lines used in this study were CCA1::LUC [75], LHY::LUC [76], PRR9::LUC [27], TOC1::LUC [77], PRR7::LUC, and FKF::LUC (Kay lab). Arabidopsis seeds were surface sterilized, plated on 1X Murashige and Skoog (MS) medium without sucrose, and stratified in the dark for 3 nights at 4 °C. Seeds were grown at constant 22 °C with ~90 μmol photons·s^−1^·m^−2^, in 12 h light and 12 h dark cycles (LD) for 7 d. Plates were sprayed with 1 mM D-luciferin (Goldbio, LUCK-100, St Louis, USA), and imaged every 1 h with a 5 min exposure for 5 d using a digital CCD camera (Andor iKon-M 934, Oxford Instruments, UK). On the third day (ZT56, 3 h before the night), plates were flooded and seedlings were submerged with 35 mL of water to fully cover the seedlings. Both control and submerged plates were imaged for an additional 3 days. Imaging results were processed using ImageJ software and analyzed with BioDare2 using the MESA analysis method [78].

## Data Availability

The sequences reported in this paper have been deposited in the National Center for Biotechnology Information (NCBI) Gene Expression Omnibus (GEO) database and can be accessed through GEO Series accession GSE215159.

## Supplementary Materials

The supporting information can be downloaded at: https://www.mdpi.com/article/10.3390/ijms24108555/s1.
