# Supplementary figures and images for: Submergence Stress Alters the Expression of Clock Genes and Configures New Zeniths and Expression of Outputs in Brachypodium distachyon
Source: Int J Mol Sci. 2023 May 10;24(10):8555. doi: 10.3390/ijms24108555 (PMC10218231; doi:10.3390/ijms24108555)

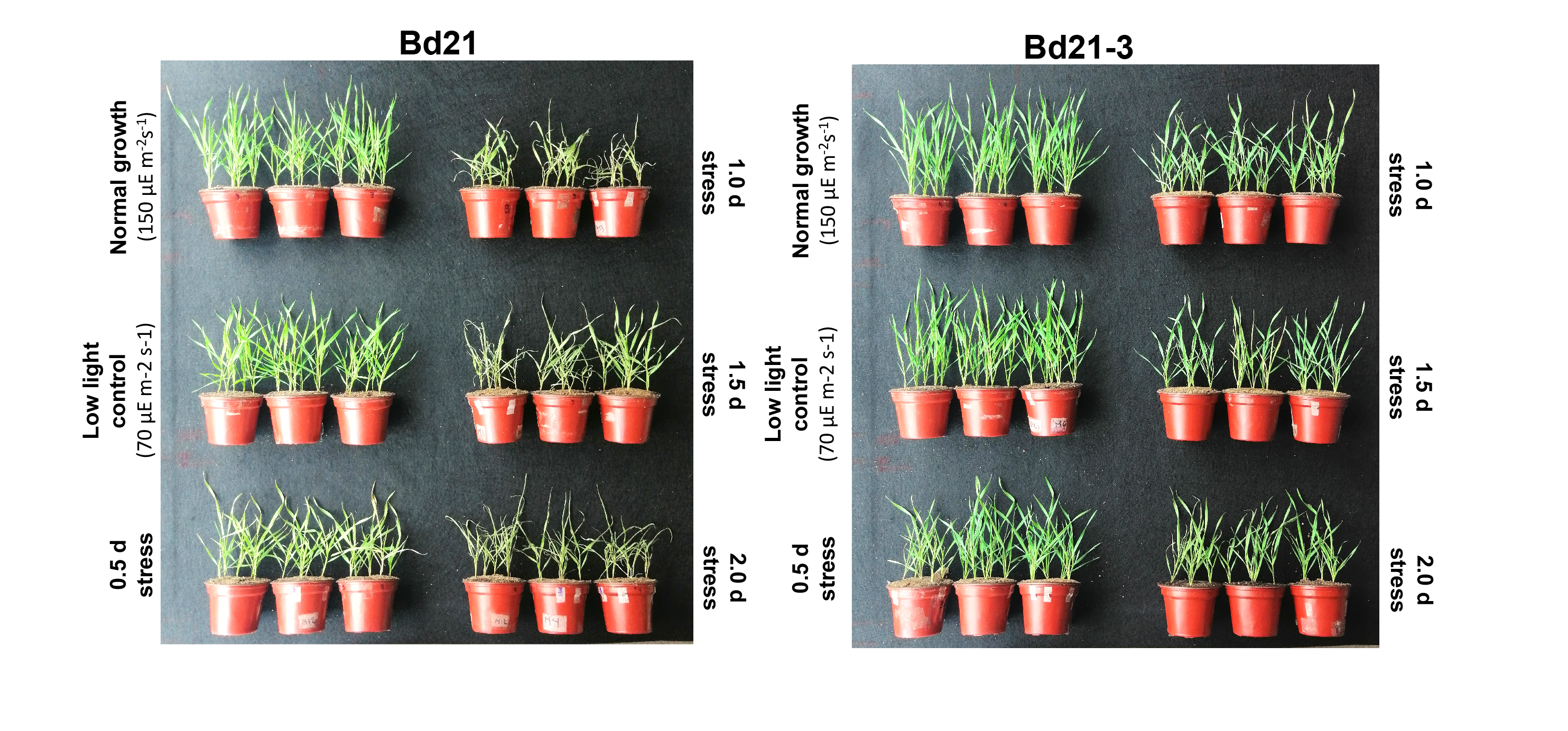

Supplement: Supplementary file 1 [file ijms-24-08555-s001.zip › supplemental_Figure_S1.tif]

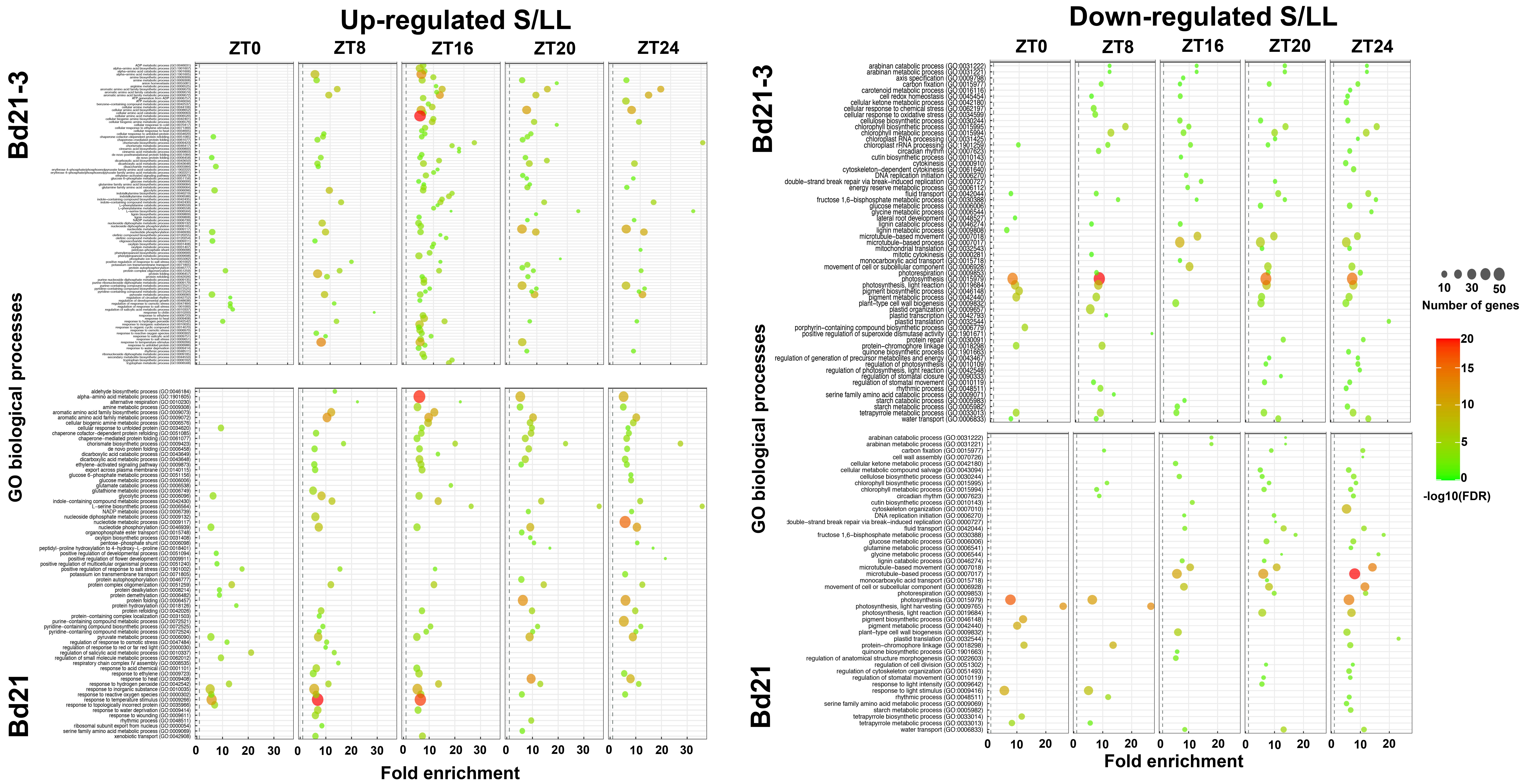

Supplement: Supplementary file 1 [file ijms-24-08555-s001.zip › supplemental_Figure_S2.tif]
